# Non-Coding RNA-Based Therapeutic Strategies in Triple-Negative Breast Cancer: A Systematic Review

**DOI:** 10.3390/ijms27041882

**Published:** 2026-02-15

**Authors:** Giovana Prado Scaratti, Inaiê Maiala de Almeida Miranda, Emanuelle Nunes-Souza, Mayara Oliveira Ruthes, Daiane Rosolen, Aline Simoneti Fonseca, Luciane Regina Cavalli

**Affiliations:** 1 Instituto de Pesquisa Pelé Pequeno Príncipe, Av. Silva Jardim, 1632, Curitiba 80250-060, Brazil; 2 Faculdades Pequeno Príncipe, Av. Iguaçu, 333, Curitiba 80230-020, Brazil; 3 Department of Oncology, Lombardi Comprehensive Cancer Center, Georgetown University, Washington, DC 20007, USA

**Keywords:** TNBC, non-coding RNA, MicroRNA, long non-coding RNA, circular RNA, therapy, pre-clinical studies

## Abstract

Triple-negative breast cancer (TNBC) is characterized by marked clinical and molecular heterogeneity, which underlies the limited success of currently available targeted therapies and results in most patients relying on cytotoxic chemotherapy. This therapeutic gap underscores the pressing need for novel therapeutic approaches, in which non-coding RNAs (ncRNAs) have emerged as promising candidates. In this systematic review, 35 pre-clinical studies published between 2020 and 2025 were analyzed to evaluate the therapeutic potential of targeting ncRNAs in TNBC, including miRNAs, lncRNAs, and circRNAs. The original articles employed in vivo tumor models to assess the therapeutic response of ncRNA expression modulation, using miRNA mimics, antagomiRs, ASOs, shRNAs, and siRNAs integrated into advanced targeted delivery systems, such as nanoparticles and exosomes. According to the selected studies, 28 specific ncRNAs were identified as actionable molecular targets. Modulation of these molecules consistently resulted in tumor growth suppression, metastasis inhibition, and restoration of sensitivity to standard chemotherapeutic agents. Collectively, the pre-clinical evidence presented in these studies positions ncRNA-based therapies as innovative, promising, and potentially effective strategies for advancing TNBC treatment.

## 1. Introduction

Triple-negative breast cancer (TNBC), characterized by the absence or low levels of ER, PR, and HER2 receptors, accounts for 15–20% of all breast cancer cases and is the most aggressive breast cancer subtype [1,2]. The lack of these surrogate markers makes the corresponding targeted therapies ineffective, resulting in most of the TNBC patients relying on systemic chemotherapy and radiotherapy [3,4].

Recently, however, the TNBC therapeutic landscape has expanded beyond conventional cytotoxic regimens. Novel therapies include immune checkpoint, PARP, androgen receptor, PI3K/AKT/mTOR inhibitors, antibody drug conjugates (ADCs), and epigenetic therapies [5,6]. Increasing interest has also been directed toward RNA-based approaches targeting histone modifiers and non-coding RNAs (ncRNAs) [7,8,9].

NcRNAs, such as microRNAs (miRNAs), long non-coding RNAs (lncRNAs), and circular RNAs (circRNAs), have emerged as promising therapeutic targets due to their critical roles in regulating gene expression, tumor progression, and therapy resistance in TNBC. These molecules can modulate oncogenic and tumor-suppressive pathways by regulating gene expression and signaling cascades, offering novel possibilities for precision medicine-based interventions [10,11,12].

Considering the complex molecular heterogeneity of TNBC, comprehensive genomic and transcriptomic profiling remains essential to identify actionable molecular markers and guide personalized treatment strategies [13,14,15]. In this context, our group has a long-standing interest in the study of ncRNAs in TNBC, particularly miRNAs, through which we have identified several candidates’ miRNAs with potential clinical relevance, supporting their use as diagnostic, prognostic, treatment response predictors and therapeutic markers [16,17,18,19,20,21,22,23,24]. Equally critical is the use of robust in vivo tumor models, supported by optimized delivery systems capable of ensuring targeted RNA delivery. Such models are indispensable for validating the therapeutic relevance of candidate ncRNAs, recapitulating tumor microenvironment interactions, and accurately assessing treatment response and toxicity [25,26,27,28]. The integration of patient-derived xenografts, genetically engineered mouse models and efficient delivery systems strengthens translational relevance and accelerates the development of clinically meaningful RNA-based therapies for TNBC.

Therefore, the primary aim of this study was to identify ncRNAs with therapeutic potential in TNBC, elucidate their mechanisms of action, and assess their response to treatment in physiologically relevant in vivo models. Continued research into the functional roles of ncRNAs in TNBC, combined with high-fidelity pre-clinical modeling, are crucial to identify actionable targets for developing innovative therapeutic strategies that can more effectively improve outcomes for patients with TNBC.

## 2. Methods

### 2.1. Protocol and Registration

The present review followed the Preferred Reporting Items for Systematic Review and Meta-Analysis (PRISMA) [29,30]. The protocol of this systematic review was submitted for registration in the International Prospective Register of Systematic Reviews (PROSPERO) under registration number 1165655.

### 2.2. Eligibility Criteria

Only original articles that investigated the role of ncRNAs as therapeutic biomarkers for TNBC were selected. The therapeutic approaches investigated included the reduction/blocking (knockdown) or increase (overexpression) of ncRNA expression. Studies that demonstrated the systemic delivery of ncRNAs to restore the expression of tumor-suppressor ncRNAs or inhibitors of oncogenic ncRNAs to block their tumorigenic action were also included. Exclusion criteria were as follows: articles on ncRNA that did not report at least one of the treatment outcomes (including: survival rates, change in tumor size and weight, metastasis development) and toxicity and biosafety data; association with other therapeutic agents and/or delivery system; articles on breast cancer that did not specifically evaluate or present results on the TNBC subtype; non-original articles (reviews), editorials, letters from editors, book chapters, unpublished or non-peer-reviewed studies; articles that used small interfering RNAs (siRNAs) or short hairpin RNAs (shRNAs), that do not correspond to human RNA sequences (unless it was used to knockdown other types of ncRNA); articles that did not evaluate treatment response in vivo; and studies where ncRNA expression was modulated in cell lines before implantation in the xenograft model.

### 2.3. Information Sources and Search Strategy

The searches were conducted in February 2024, and updated in September 2025, using the electronic databases PubMed, Embase, and BVS. The search terms in the PubMed database were as follows: “untranslated RNA”, “triple negative breast cancer”, “therapy”, “in vivo” and “clinical trial” (Table S1). The articles were published between September 2020 and August 2025.

### 2.4. Study Selection and Data Collection

The articles were uploaded to Rayaan [31], an AI-powered platform created for management of systematic reviews, and duplicates were removed. In phase 1, titles and abstracts were independently screened. In phase 2, there was a full-text reading of the selected articles. Any disagreements in the selection or interpretation of the articles in phases 1 and 2 were resolved through discussion and mutual agreement.

### 2.5. Risk of Bias Assessment

SYRCLE’s Risk of Bias Tool [32] was used to assess the risk of bias. Each question was answered independently, with “Yes” indicating a low risk of bias, “No” indicating a high risk of bias, or “Unclear” for insufficient details (Table S2). A third party resolved any differences in interpretation.

### 2.6. Nomenclature of Therapeutic Approaches

To ensure consistency across the studies, the terminology of the agents modulating ncRNA expression was standardized. Synthetic oligonucleotides designed to knockdown ncRNAs (miRNAs, lncRNAs, or circRNAs) were termed antisense oligonucleotides (ASOs). Considering the prevalence of miRNA-focused studies and their established terminology, miRNA modulators were referred to as “antagomiR” and “miRNA mimic” for knockdown and overexpression, respectively. In cases lacking specific miRNA terminology, the general terms were applied. In the studies that specifically utilized siRNAs or shRNAs, their original terminology was retained to reflect their distinct structure and mechanism of action. Other nomenclatures that were not represented by the used standardization were maintained as reported, to ensure accurate representation of the methodologies.

## 3. Results and Discussion

### 3.1. Study Selection

**Figure 1:**
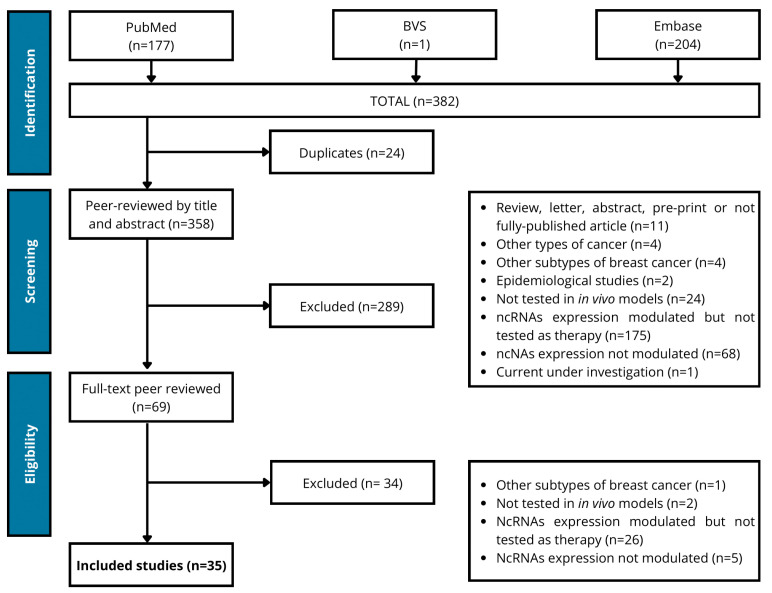
PRISMA flow diagram of study screening and selection.

A total of 382 articles were compiled from the databases that were searched according to the specific search terms used (Table S1). After removing duplicates (n = 24), 358 articles remained for initial screening, based on the evaluation of titles and abstracts. Full-text screening was performed for the 69 selected articles, of which 35 final articles were included in this review (Figure 1).

### 3.2. Risk of Bias

Across the included studies, assessment with the SYRCLE Risk of Bias Tool indicated that most domains were classified as Low Risk, indicating that the articles fulfilled and reported the methodological criteria for animal handling in general. However, some domains were classified as Uncertain Risk, reflecting omissions in the reporting of methodological details (Table S2). Most of the domains identified as uncertain were random housing of animals (D4), blinding of caregivers or investigators (D5), random selection of animals for outcome assessment (D6), blinded outcome assessment (D7), and treatment of incomplete outcome data (D8). These domains were completely underreported in seven studies [33,34,35,36,37,38,39] only. Some studies described how the allocation sequence was generated and applied (D1) (19/35), whether the groups were similar at baseline or adjusted for confounding factors (D2) (29/35), or whether the allocation was adequately concealed (D3) (17/35). Selective reporting of outcomes (D9) (16/35) was reported, contributing to additional uncertain risk classifications. Most studies appeared free of other sources of bias (D10). In general, the articles reported on the criteria for handling animals in the laboratory and pointed out the guidelines followed for each institution and country, in addition to explaining the analysis of the results pointed out by randomization, blinding of the study, and uniform animal groups.

### 3.3. General Characteristics of Selected Articles

The 35 selected articles were published between September 2020 and August 2025. Twenty-six articles were from China, six from the USA, two from South Korea, and one from Canada (Table S3).

A total of 28 ncRNAs were reported in these articles: 20 miRNAs (Table 1), seven lncRNAs, and one circRNA (Table 2). These ncRNAs were evaluated for their roles in mediating treatment response to several chemotherapeutic agents, both as single and in combined regimens. Doxorubicin was the most tested agent (n = six articles), followed by CB1954, ganciclovir (GCV), and paclitaxel (n = 2). Carboplatin, cetuximab, gemcitabine, oxaliplatin, and omacetaxine mepesuccinate were tested in one article each (Table 1 and Table 2). Combination therapies included curcumin (n = 2), TK-p53-NTR triple therapeutic gene (which combines the p53 with dual suicide gene systems (thymidine kinase (TK/ganciclovir and NTR/CB1954)) (n = 2), cold atmospheric plasma (n = 1), and pyropheophorbide-a (n = 1).

MiRNAs were reported in 25 articles and included 20 distinct miRNAs: miR-34a (n = 5 articles), miR-21 (n = 4), miR-10b (n = 2), miR-155 (n = 2), miR-100 (n = 2), miR-22, miR-105-5p, miR-139, miR-143, miR-146b-5p, miR-155-5p, miR-182-5p, miR-199-5p, miR-320c, miR-325-3p, miR-326, miR-506, miR-588, miR-606, and miR-6162 in one article each (Table 1). There were eight articles reporting seven different lncRNAs: *DARS-AS1* (n = 2), *ABHD11-AS1*, *ASBEL*, *DDIT4-AS1*, *FBXL19-AS1*, *MALAT1*, and *MILIP* in one article each. The *circDUSP16* was the only circRNA, reported (n = 1) (Table 2).

### 3.4. MiRNAs

**Figure 2:**
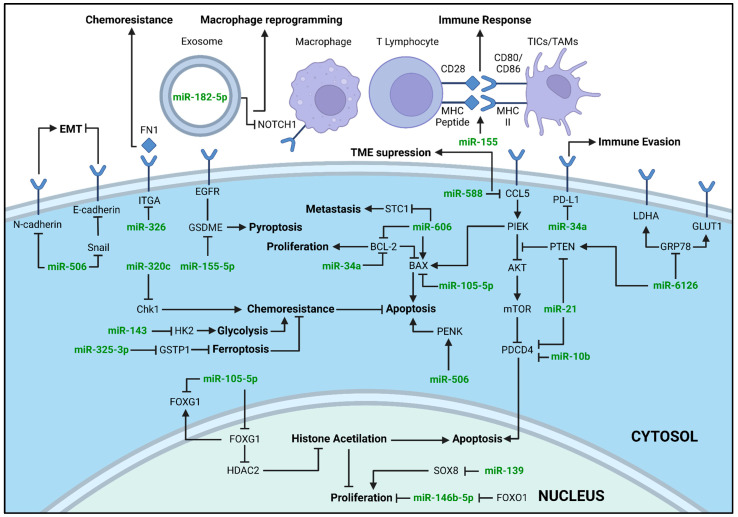
Schematic overview of miRNA-mediated therapeutic mechanisms in TNBC. MiRNAs are color-coded in green. Arrows indicate activation and blocked arrows, inhibition. The resulting biological effect of the miRNAs in TNBC cells are highlighted in bold. Image created using *BioRender* (BioRender.com), accessed on 14 December 2025.

Due to their diverse biological functions and tissue-specific expression patterns, miRNAs are a class of ncRNAs with significant clinical interest for the development of novel diagnostic, prognostic, and therapeutic methods [68]. Of the 35 selected studies, 26 reported the therapeutic potential of modulating the expression of 20 miRNAs in TNBC in vivo models (Table 1, Figure 2). In these articles, 12 miRNAs (miR-34a, miR-100, miR-139, miR-143, miR-146b-5p, miR-155, miR-320c, miR-325-3p, miR-506, miR-588, miR-606, and miR-6162) were reported with their expression upregulated by using miRNA ectopic expression approaches (miR-mimics). One miRNA was upregulated by using a virus expressing pre- or pri-miR-199a-5p, and another by inducing miR-326 expression with doxycycline. Five miRNAs (miR-10b, miR-21, miR-22, miR-155-5p, and miR-182-5p) were silenced using antagomiRs and two miRNAs (miR-21 and miR-105-5p) using non-specified miRNA inhibitors. Combination therapy was tested in 13 of the miRNA articles, with the following agents: CB1954 [33,41], cetuximab [51], cold atmospheric plasma [49], curcumin [50], doxorrubicin [37,40,42,56], ganciclovir [33,41], gemcitabine [48], oxiplatin [54], paclitaxel [55], pyropheophorbide-a [39], and TK-p53-NTR triple therapeutic gene [33,41].

#### 3.4.1. miRNA Mimics

MiRNA mimics are synthetic oligonucleotides constructed to copy the function of an already existing miRNA. Of the 12 miRNAs that were upregulated using miRNA mimics, miR-34a was the most cited. The miR-34a family of miRNAs plays an important role in tumor suppression in various types of cancer, including breast cancer, where its high expression is associated with less aggressive tumor behavior [69,70]. The study by Zhao et al. [44] evaluated the therapeutic potential of miR-34a using a zeolitic metal–organic framework (MOF) nanoparticle for delivery. The zeolitic imidazolate framework 8 (ZIF-8) MOF protected miRNA mimics from RNase degradation in blood, and, once inside the cytoplasm, released miR-34a mimics by decomposing itself and releasing Zn+. Treatment with miR-34a-m@ZIF-8 complex was shown to inhibit B-cell lymphoma 2 gene (*BCL-2*) expression and reduce tumor growth, in comparison to controls. In addition to protecting miRNA mimic and improving delivery to cells, ZIF-8 was able to induce tumor cell apoptosis by releasing reactive oxygen species [44]. The second study to use miR-34a mimic as a therapy approach for TNBC was conducted by Deng et al. [45]. These authors showed that intra-tumoral injection of miR-34a in mice could downregulate the immune checkpoint Programmed Cell Death Ligand 1 (*PDL-1*) gene. Compared to the PBS-treated group, the miR-34a mimic-treated group resulted in tumor volume reduction [45]. Additionally, Han et al. [42] indicated that miR-34a functions as a tumor inhibitory factor, inhibiting the expression of Cluster of Differentiation 44 (CD44) and BCL2 apoptosis regulator mRNAs. Han et al. [34] developed a nonviral vector PEI-SPDP-Man (PSM) by connecting mannitol (Man) to branched polyethylenimine (PEI) using a disulfide bond. The vector entered tumor cells via endocytosis, reducing miR-34a degradation. Mice treated with PSM/miR-34a showed smaller tumor volume and size compared to mice treated with saline or PEI/miR-34a. Treatment with PSM/miR-34a was also capable of inhibiting tumor cell proliferation and inducing apoptosis [34]. Another treatment approach was conducted by Duan et al. [37], where miR-34a mimic was combined with doxorubicin. Both therapeutics were loaded into redox-responsive diselenide mesoporous silica nanoparticles (MONs) and then enveloped by cancer cell membrane-derived vesicles (CM), with the final product named MONSe@Dox@miR-34a@CM. Experiments in xenografted mice revealed that MONSe@Dox@miR-34a@CM had the smallest tumor volume compared to control groups. Moreover, this treatment also showed a higher apoptotic rate, necrosis, and lower cell proliferation. Treatment of MDA-MB-231 mammospheres with MONSe@Dox@miR-34a@CM led to a reduced expression of Aldehyde Dehydrogenase 1 Family Member A3 (*ADLH1A3*), *CD44*, Wnt Family Member 7B1 (*Wnt7b*), and Transcription Factor 4 (*TCF4*) compared to non-treated cells. This finding suggests that the treatment controls cell proliferation by regulating the expression of these proteins [37]. Lastly, Xiong et al. [39] developed a targeted delivery system using aptamer-modified liposomes (Apt-LP), with incorporated pyropheophorbide-a (pyro), to transport miR-34a mimic to tumor cells. The incorporation of aptamers enabled the specific targeting of cancer cells. Upon near-infrared (NIR) irradiation (L+), the pyro molecules located on the liposome’s external surface were activated, generating ROS, leading to the disruption of the liposome and lysosomes, thereby releasing the mimics inside the cell and protecting them from lysosomal degradation. In vitro experiments showed that treatment with Apt-LPR (L+) suppressed *BCL-2* and *PDL-1* expression in both MDA-MB-231 and 4T1 cell lines. Mice treated with Apt-LPR (L+) had the longest survival among all groups, accompanied by suppressed growth and reduced volumes in both local and distal tumors, an increased number of apoptotic cells, reduced blood vessel formation, and the absence of lung metastasis. Also, treatment with Apt-LPR (L+) reversed the immunosuppressive tumor microenvironment by suppressing *PDL-1* expression and inducing immunogenic cell death, which promoted CD8+ T cell tumor infiltration, dendritic cell (DC) maturation and macrophage M1 polarization. Similar results in tumor growth and cell apoptosis were also seen in zebrafish tumor models [39]. The biosafety of the described delivery systems above was mainly evaluated by measuring mice’s body weight, analyzing biomarkers in serum associated with organ function, and by assessing pathological damage in the main organs. All treatments showed similar results compared to PBS-treated mice, demonstrating the lack of adverse effects [34,39,44,45]. 

MiR-100 is a tumor suppressor that targets genes associated with cell growth and stress response, and it is frequently dysregulated in different types of cancer [71,72]. Liu et al. [41]’s study in TNBC lung metastasis evaluated the combination of miR-100 mimic, miR-21 antagomiR, CGV, CB1954, and a fusion of type 1 thymidine kinase (TK), nitroreductase (NTR), and TP53 (TK-p53-NTR), with the addition of miR-10b antagomiR. TK-p53-NTR and synthetic oligonucleotides were loaded to gold nanoparticles (AuNPs) coated with chitosan–B-cyclodextrin (CS-CD) and urokinase plasminogen activator (uPA). Intranasal delivery of pAuNS@TK-p53-NTR-miRs to TNBC lung metastasis mice xenografts, together with intraperitonial injection of GCV and CB1954, showed improvement in survival rates and inhibition of tumor growth, compared to controls. Despite improving survival, treatment with AuNS-Tk-p53-NTR-miRs did not inhibit lung metastasis, which was seen in all test groups. In vitro delivery of anti-miR-10b, anti-miR-21, and miR-100 mimic upregulated the expression of Homeobox D10 (*HOXD10*) and phosphatase and TENsin Homolog Deleted on Chromosome 10 (*PTEN*) [41]. Kumar et al. [33] also studied the therapeutic potential of miR-100 mimic combined with ganciclovir (CGV), CB1954, miR-21 antagomir, and TK-p53-NTR. Nucleotides were encapsulated in polymer nanoparticles made from poly (lactic-co-glycolic acid) (PLGA), poly (ethylene glycol) (PEG), and PEI (PLGA-PEG-PEI) to ensure precise delivery to tumors upon ultrasound destruction of microbubbles. Asyngeneic 4T1 tumor (expressing FLuc-EGFP) model in immunocompetent mice treated with leaded PLGA-PEG-PEI + US-MB increased the treatment effects, extended survival rates and presented smaller tumors compared to controls or passive delivery. The combination of miRNAs leads to effective sensitization of tumor cells to TK-p53-NTR-mediated prodrug (GCV and CB1954) therapy and enhanced apoptosis, resulting in improved therapeutic effects as shown by the reduction in tumor growth and improvement in animal survival rates [33]. Regarding toxicity, both studies found that mice body weight decreased during treatment. Also, oligonucleotide vehicles were found in other organs (heart, spleen and lungs in Kumar et al. study and heart and brain in Liu et al. study), although H&E staining did not reveal any tissue damage [33,41].

Two articles identified testing miR-155 as an intervention for TNBC in vivo models: Jing et al. [35] and Li et al. [50]. In the study of Jing et al., the miRNA was delivered through a nanocomplex of (ethylene glycol)-carboxydimethyl maleate-poly(ethyleneimine)-peroxalate ester-poly (ε-caprolactone) grafted with mannose moieties (PEG-CDM-PEI[Man]-ox-PCL) [35]. These miR-155 complexes induced lower Antigen Kiel 67 (Ki67) positive cell with higher apoptotic rates, and reprogrammed tumor-infiltrating dendritic cells (TIDCs) and tumor-associated macrophages (TAMs), by upregulating the expression of T-Lymphocyte Activation Antigen CD80 (CD80), cluster of differentiation 86 (CD86) and Major Histocompatibility Complex Class II (MHC II) [35]. CD80 and CD86 are surface proteins that interact and activate transmembrane proteins, such as CD28 and CTLA-4, on T cells [73]. TIDCs and TAMs are antigen-presenting cells which play an important role in tumor immunity [74,75]. In vivo experiments also showed anti-tumor immune response by efficient intra-tumoral TIDCs, and TAM reprogramming reduced tumor volume and weight and lung metastasis, with a longer survival rate compared to the control group. Moreover, Jing et al. evaluated the nanocomplex biodistribution, and showed its accumulation in the tumors, proving its targeting specificity capability [35].

Li et al. [50] used a reactive oxygen species/glutathione dual-sensitive nanoparticle to deliver miR-155 and curcumin (CUR/miR155@DssD-Hb NPs) through tail vein injections. In the tumor cells, this nanoparticle system reduced cell proliferation and migration, induced apoptosis, stimulated ROS generation and the secretion of High Mobility Group Box 1 (*HMGB1*) and Adenosine Triphosphate (ATP) [50]. Both are damage-associated molecular pattern (DAMPs) molecules which act in immunogenic cell death [76]. In in vitro studies with DCs, CUR/miR155@DssD-Hb NPs led to their maturation and a higher expression of CD11c+ (CDs makers) and MHC-II+ (antigen-presenting protein) [50]. DCs are antigen-presenting cells that migrate from the tissue to the lymph nodes and activate cytotoxic T lymphocytes through antigen-presenting protein, such as MHC-II [74]. In the mice, the delivery suppressed tumor growth and weight, and improved survival rate [50]. Li et al. evaluated lung metastasis by analyzing the number of nodules macro- and microscopically. The CUR/miR155@DssD-Hb NP group had significantly lower levels of lung metastasis and in tumor-draining lymph nodes, the number of matured DCs and IFN-γ+ CD8+ T cells were higher. Li et al. also showed that the nanoparticles successfully delivered miR-155 and curcumin to the TNBC cells in in vitro and in vivo models [50].

Silencing of miR-139 is a common phenomenon in solid tumors, and restoring its expression can inhibit tumor growth and induce cell death [77,78]. Dong et al. [47] investigated the tumor suppressing action of miR-139 using miRNA mimic in TNBC mouse models. Compared to the control group, mice treated with the miR-139 mimic presented reduced tumor volumes and weights. Treatment with miR-139 mimics suppressed SRY-Box Transcription Factor 8 (*SOX8*) expression in the HCC1806 and BT549 cell lines. Furthermore, in vivo treatment with the miR-139 mimic and SOX8 pcDNA3.1 resulted in tumors with higher volume and weight compared to treatment with the miR-139 mimic alone [47].

Another miRNA frequently downregulated in tumors is miR-143, a tumor suppressor that can inhibit glycolysis, reducing energy available for tumor progression [79]. Glycolysis also plays a role in chemotherapy resistance in cancer, reducing the uptake of drugs, such as gemcitabine (GEM), by downregulating adenosine transporters [80]. Xi et al. [48] used miR-143 mimic to reverse GEM resistance (GEM-R) in TNBC mice models. Although miR-143 mimic combined with GEM could inhibit tumor growth in GEM-R mice, more effective results were seen in GEM-R with human equilibrative nucleoside transporter 1 (*hENT1*) overexpression (GEM-R-hENT1). Also, suppression of hexokinase 2 (*HK2*) was seen in GEM-R-hENT1 mice treated with miRNA mimic and GEM, suggesting that miR-143 inhibited glycolysis by targeting *HK2*, and reversing GEM-R with hENT1 assistance [48].

Inflammation is a common process of most types of cancer, as it promotes immuno-suppression, providing an ideal environment for tumor progression [81]. Dai et al. [49] showed that combining cold atmospheric plasma (CAP) with miR-146b-5p mimic reduced inflammation. Subcutaneous injections of mimics and CAPs near tumor sites in xenografted mice suppressed tumor growth, in comparison to control mice. In vitro and in vivo experiments showed a role for CAP in the maturation and stabilization of miR-146-5p by elevating Methyltransferase 14 (METTL14) levels and promoting interaction between the DGCR8 Microprocessor Complex Subunit (DGCR8) and miR-146b-5p. Furthermore, CAP silenced Forkhead Box 1 (FOXO1) and SR-Related CTD-Associated Factor 11 (SCAF11) expressions [49].

Lim et al. [54] showed that the dual delivery of miR-320c (by intra-tumoral injections) and the drug oxaliplatin (by intraperitoneal injection) reduced the expression of CheckPoint Kinase 1 (CHK1), a checkpoint kinase involved in DNA damage response (DDR) and cell cycle [82], that can promote tumor growth and may contribute to anticancer therapy resistance [83]. MiR-320c/oxaliplatin suppressed the expression of *CHK1*, resulting in apoptosis reduction and oxaliplatin-induced DDR inhibition in TNBC cells [54]. In in vivo models, dual therapy also led to an increase in the expression of Phosphorylated Histone H2AX (γ-H2AX) and cleaved caspase-3 (CASP3), increasing apoptosis, which supported that the miRNA mimic injection-enhanced oxaliplatin treatment in TNBC [54].

In their 2025 study, Wang et al. [55] demonstrated that restoring levels of the miR-325-3p can effectively overcome paclitaxel resistance in TNBC. The authors showed that delivering miR-325-3p mimics in combination with paclitaxel suppresses tumor growth and proliferation by directly targeting and downregulating Glutathione S-Transferase P 1 (GSTP1), a key protein associated with chemoresistance [55,84]. The inhibition of *GSTP1* not only restored sensitivity to paclitaxel, but also induced ferroptosis, a non-apoptotic form of regulated cell death that is part of the innate tumor-suppressor mechanism [85], providing a mechanism to eliminate resistant cancer cells.

Liu et al. [57] demonstrated that encapsulating the miR-506 within gelatin nanospheres (miR-506 mimic + lipo 2000-loaded GNs), delivered through intra-tumoral injection, significantly enhanced its therapeutic efficacy against TNBC. This delivery system enabled efficient targeting and upregulation of Proenkephalin (*PENK*) gene, leading to suppression of the Extracellular Signal-Regulated Kinase/FOS proto-oncogene (*ERK*/*FOS*) signaling pathway, an axis associated with tumor proliferation and invasiveness [57,86]. In in vivo experiments, miR-506 mimic inhibited cell viability, colony formation and migration, and led to a significant increase in Epithelial Cadherin protein (E-Cadherin) expression and decrease in Neural Cadherin (N-Cadherin) and Snail [57]. These proteins are involved in the epithelial–mesenchymal transition (EMT), a process that enables the formation of cancer metastasis and is associated with drug resistance [87]. PENK mRNA and protein expression were also increased in vivo after miR-506 mimic [57]. PENK has already shown to be associated with cancer metastasis in osteosarcoma and in breast cancer [88,89]. The mice treated with miR-506-loaded nanospheres presented with reduced tumor growth, weight, and volume [57]. Vimentin (VIM), ERK, and FOS expression decreased in vivo, while E-cadherin increased. The mice’s histological evaluation showed TNBC necrosis and apoptosis. Liu et al. also showed miR-506-loaded nanosphere delivery, which led to a decrease in lung metastasis which did not cause toxicity in the lung tissue [57].

In the study conducted by Zhang et al. [58], the delivery of miR-588 via cRGD-modified exosomes (cRGD-Exos/miR-588), through tail vein injection, effectively enhanced its therapeutic impact on TNBC. The engineered exosome system downregulated C-C Motif Chemokine Ligand 4 (CCL5) and the Transforming Growth Factor-Beta (TGF-β), mediators involved in the tumor microenvironment [58,90]. MiR-588 delivery successfully remodeled the immunosuppressive tumor microenvironment, by regulating *CCL5* to alter TAM polarization, and by enhancing NK cells abilities, reaching anti-tumor efficacy [58]. Functionally, this immunomodulatory shift leads to a significant inhibition of tumor growth. cRGD-Exos/miR-588 also demonstrated effective accumulation at tumor sites, indicating favorable biodistribution. In addition, no mortality nor severe weight loss were observed in the intervention group and H&E staining revealed no significant damage to major organs, collectively supporting the favorable in vivo safety [58].

Choi et al. [36] evidenced that at the molecular level, miR-606 exerts its effects primarily by directly binding and repressing Stanniocalcin 1 (STC1), a glycoprotein previously implicated in promoting tumor growth and metastatic dissemination [91,92]. Suppression of STC1 was previously shown to trigger downstream alterations in key regulatory proteins, such as Proliferating Cell Nuclear Antigen (PCNA), BCL-2, and BCL2-associated X (BAX); PCNA, a marker of DNA synthesis and cellular proliferation, and BCL-2, an anti-apoptotic protein, were shown to be reduced, thereby diminishing the survival capacity of cancer cells and BAX, a pro-apoptotic effector, which is to be increased, showing that STC1 repression shifts the cellular balance toward apoptotic cell death [93,94]. Functionally, these molecular changes translate not only into suppressed tumor volume and weight in vivo but also into a marked decrease in lung metastasis [36]. IHC analysis of the TNBC in vivo showed fewer tumor cells, increased expression of BAX, and lower expression of STC1, PCNA, and BCL-2 [36].

Wang et al. [59] demonstrated that miR-6126 exerted a potent suppressive effect on the metabolic and oncogenic programs characteristic of TNBC. MiR-6126 mimic delivered through intra-tumor injection in nude mice inhibits the expression of Warburg effect-associated signaling proteins such as Glucose-Regulated Protein 78 (GRP78), Hypoxia-Inducible Factor 1-Alpha (HIF1α), Glucose Transporter Type 1 (GLUT1), and Lactate Dehydrogenase A (LDHA) but induces *PTEN* expression. MiR-6126 disrupted both the Warburg effect and aberrant mitochondrial dynamics, thereby impairing the metabolic reprogramming required for rapid tumor proliferation. This multifaceted regulatory activity in vivo induces apoptosis, and inhibits metastasis and tumorigenesis, leading to a marked decrease in tumor growth [59].

MiRNA mimics were not the only strategy used to overexpress tumor-suppressor miRNAs. St–Cyr et al. [53]’s study used modified vesicular stomatitis virus (VSVd51) that expressed pre-miR-199a-5p to treat TNBC mice xenografts. In vitro, miR-199a-5p overexpression inhibits Zinc Finger E-Box-Binding Homeobox 1 (ZEB1) expression, suggesting a regulatory role in EMT. Despite in vitro results and previous evidence in the literature of anti-tumor miR-199a-5p function [95], miR-199a-5p overexpression did not affect the expression of EMT-associated genes in vivo, neither did it affect tumor growth nor mice survival [53]. Another strategy was tested in Assidicky et al.’s study [56], where they induced miR-326 overexpression in nude mice with doxorubicin-sensitive/resistant xenografts treated with doxorubicin [56]. The authors demonstrated that elevated miR-326 levels in three TNBC cell lines (MDA-MB-231, MDA-MB-157, and MDA-MB-436) effectively downregulated the Integrin Subunit Alpha 5 (*ITGA5*), a key component of the Fibronectin (FN1) receptor complex. This suppression was accompanied by reduced phosphorylation of Focal Adhesion Kinase (FAK) at Y397 and v-*SRC* Sarcoma Viral Oncogene Homolog (SRC) at Y416, indicating inhibition of downstream integrin-mediated signaling pathways associated with cell survival, adhesion, and chemoresistance. In vivo, the inhibition of *ITGA5* gene expression driven by miR-326 overexpression significantly enhanced tumor sensitivity to doxorubicin and delayed tumor growth [56].

#### 3.4.2. AntagomiRs

MiRNAs also drive tumorigenesis when they function as oncomiRs [96,97,98]. In such cases, miRNA inhibitors, such as ‘antagomirs’, offer an efficient strategy for silencing these oncogenic miRNAs and represent a promising therapeutic approach [99]. One of these miRNAs is miR-21, which has been associated with anti-apoptotic and chemoresistance activities in breast cancer [100,101,102]. Four articles selected in this review cited the silence of miR-21 as a potential treatment for TNBC [33,40,42]. This miRNA can inhibit the expression of several key suppressor tumor genes, such as Programmed Cell Death 4 Protein (*PDCD4*) [33,40], *PTEN* [41,42], and *TP53* [42]. In the study of Liu et al. [40], a cancer cell–platelet fusion membrane vesicle (CPMV) was developed using a microfluidic platform for site-specific delivery of two antisense miRNAs, anti-miRNA-10b and anti-miRNA-21. An intravenous delivery method of these anti-miRNA-loaded CPMVs was administered in mice-bearing xenografts of MDA-MB-231 tumors. A higher tumor growth was observed in the control group compared to the animals treated with anti-miRNAs-CPMVs with or without the combination of DOX. In both groups, loss of body weight was observed in the animals. Interestingly, the expression analysis of anti-miR-21 by RT-qPCR demonstrated its expression levels in the heart (55%), spleen (320%), and lungs (51%), compared to the levels detected in the tumor sites (100%). The study of Kumar et al. [33] demonstrated that ultrasound microbubble-mediated co-delivery of antagomiR-21, miR-100, and TK-p53-NTR is an efficient therapeutic strategy for TNBC. These authors, using PLGA-PEG-PEI nanoparticles in mouse models, reported significantly improved tumor regression and longer survival rates in animals with syngeneic hepatocellular carcinoma and TNBCs, compared with controls, upon the delivery of ganciclovir and CB1954 [33]. A similar therapeutic strategy was tested in Liu et al.’s study [41], with the addition of antagomiR-10b. In this study, AuNPs were used to deliver TK-p53-NTR-miR TNBC lung metastasis in xenograft models, resulting in an increase in survival rates and decreased tumor growth. In vitro, HOXD10 and PTEN expression were upregulated when treated with antagomiR-10b, antogomiR-21, and miR-100 [41]. The last miR-21 study, conducted by Chen et al. [42], also used DOX, but in a new nanosystem based on amphiphilic phosphorus dendron (1-C12G1) micelles to co-deliver miR-21 inhibitor (miR-21i) and this chemotherapeutic agent for TNBC. MDA-MB-231 cells treated with 1-C12G1/miR-21i showed a reduced cell viability with an increase in DOX concentration,; however, free DOX, 1-C12G1@DOX and 1-C12G1@DOX/miR-21i also induced cytotoxicity to MDA-MB-231 cells, which was observed in a DOX concentration-dependent fashion. When this combination was tested in a TNBC orthotopic model, free DOX, 1-C12G1/miR-21i, 1-C12G1@DOX or 1-C12G1@DOX/miR-21i polyplexes led to higher tumor growth inhibition compared with 1-C12G1 without DOX. In the group of 1-C12G1@DOX/miR-21i polyplexes, the tumor cells display the highest expression levels of *BAX*, *PTEN* and p53 proteins among all groups, which led to the increase in apoptosis. Free DOX toxicity to mice led to weight loss, damage to main organs, and death. However, 1-C12G1@DOX or 1-C12G1@DOX/miR-21i polyplexes showed the opposite effect. Therefore, the authors pointed out that the 1-C12G1@DOX/miR-21i polyplexes can be used to co-deliver both DOX and miR-21i to improve tumor inhibition efficacy and reduce the systemic toxicity of DOX. This occurs via rapid drug release at slightly acidic tumoral pH and the passive permeability and retention effect [42].

Other oncogenic miRNAs identified with therapeutic potential in this review were miR-22 [43], miR-105-5p [46], miR-155-5p [51], and miR-182-5p [52]. MiR-22 upregulation can promote EMT, tumor invasion, and metastasis in hormone-responsive breast cancer [103,104]. Panella et al. [43] reported that miR-22 overexpression in human TNBC xenografts led to decreased overall survival and increased metastatic dissemination to the lungs. Anti-mir-22 administration reversed these effects, leading to longer overall survival, and suppression of metastatic dissemination and tumor growth [43].

MiR-105-5p is also highly expressed in TNBC tumors, where it is associated with poor prognosis [46,105]. In the study of Wang et al. [46], higher expression of miR-105–5p was observed in the MDA-MB-231 and BT-549 cells. These cells also contained high expression levels of Histone Deacetylase 2 (HDAC2) but lower levels of the Forkhead Box Protein G1 (FOXG1). The transfection with the miR-105–5p inhibitor increased the protein levels of the apoptosis-promoting factor BAX and inhibited the levels of BCL-2. Interestingly, the silencing of *FOXG1* gene (sh-FOXG1) weakened the effect of the miR-105-5p inhibitor, while the treatment with garcinol strengthened the effect of sh-FOXG1, promoting BCL-2 protein expression and decreasing BAX. The same effect was observed in nude mice models, where the inhibition of miR-105–5p decreased tumor cell proliferation in animals, and the knockdown of *FOXG1* weakened the suppressive effect of the miR-105–5p inhibitor on tumor cell proliferation and promoted cancer cell proliferation [46].

MiR-155-5p potential in TNBC treatment was investigated in the study of Xiu et al. [51], in combination with the antibody cetuximab. This miR-155-5p was shown to be overexpressed in TNBC with Epidermal Growth Factor Receptor positivity (EGFR+). In Xiu et al. [51]’s study, the authors showed a synergetic action with the silence of miR-155-5p (antagomiR) and treatment with cetuximab, which led to cell death by pyroptosis and apoptosis. Apoptosis is the main mechanism of cell death triggered by this antibody cetuximab [51,106] and pyroptosis is a cell death mechanism associated with Gasdermin E (*GSDME*), a direct target of miR-155-5p [51,107]. In vivo, the combination of cetuximab and miR-155-5p antagomir reduced tumor volume and weight, compared to treatment with cetuximab alone. This combination treatment also suppressed cell proliferation in tumor tissues, induced cell apoptosis, upregulated the expression of GSDME and cleaved Caspase-1 (CASP1), and downregulated the expression of p-EGFR. These findings indicated that the downregulation of miR-155-5p enhanced the anti-tumor effect of cetuximab in TNBC cells in vivo [51]. Finally, the last oncomiR identified in this review with TNBC treatment potential was miR-182-5p. In Li et al. [52] study, a cell-derived xenograft (CDX) BALB/C nude-mouse model was created to demonstrate that exosome-derived miR-182-5p from TNBC cells can reprogram M2 macrophage polarization through direct combination with Neurogenic Locus Notch Homolog Protein 1 (*NOTCH1*), thereby enhancing breast cancer progression. The results showed that tumor volume, weight, and proliferation were significantly reduced in miR-182-5p-antagomir-treated mice. In addition, M2 macrophage polarization was notably downregulated, demonstrating its impact on suppressing tumor growth and limiting the pro-tumorigenic effects of M2 macrophage polarization [52].

Collectively, these studies, using miRNA mimics and silencers, in combination with chemotherapy, targeted therapies, and tumor-specific delivery systems, highlight the potential of miRNA modulation as promising therapeutic strategies against TNBC cancer cells.

### 3.5. LncRNAs and circRNA

**Figure 3:**
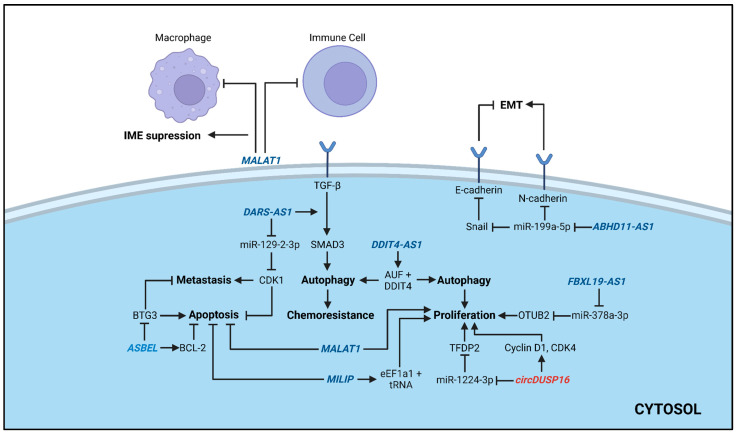
Schematic overview of lncRNAs and circRNA-mediated therapeutic mechanisms in TNBC. LncRNAs in blue, and circRNA in red. Arrows indicate activation and blocked arrows, inhibition. The resulting biological effect of the ncRNAs in TNBC cells are highlighted in bold. Image created using *BioRender* (BioRender.com), accessed on 14 December 2025.

LncRNAs play a critical role in several biological activities and are widely considered as potential drug design targets due to their high tissue specificity [108,109]. Among the 35 selected in vivo studies in this systematic review, eight articles reported seven silenced lncRNAs with therapeutic potential in TNBC. Three silenced lncRNAs, *ASBEL*, *MALAT1* and *MILIP*, were reported by antisense oligonucleotides (ASOs), two (*DARS-AS1* and *DDIT4-AS1*) by siRNA and two (*ABHD11-AS1* and *FBXL19-AS1*) by shRNA (Table 2, Figure 3). The combination therapy used in lncRNAs studies were carboplatin [65], curcumin [61], doxorubicin [63,66], omacetaxine mepesuccinate (INN) [66], and paclitaxel [64].

#### 3.5.1. ASOs

ASOs can directly target specific genes or transcripts by complementary base pairing and can therefore be designed exclusively based on gene sequence information [110]. The first of the three studies that used ASO to silence lncRNAs was the study conducted by He et al. [61] that indicated the *ASBEL* lncRNA as an oncogene that suppresses the tumor-suppressor B-Cell Translocation Gene 3 (*BTG3*) that also regulates *BCL-2* and the Cellular Mesenchymal–Epithelial Transition gene (*c-MET)* in TNBC. The authors pointed out that Antago3, a synthetic peptide, can inhibit *ASBEL* and that the agent curcumin can inhibit the canonical Wnt/β-catenin pathway, which is a target of *ASBEL*. They also demonstrated that self-assembled nanocomplexes based on the bioactive polyelectrolytes, hyaluronic acid (HA) and cationic polysaccharide of chitosan (CS), can be used for the co-delivery of Antago3 and curcumin (CANPs), enabling the collaborative modulation of *ASBEL* expression for synergetic TNBC therapy. In treated mice, this combination showed significantly higher tumor and metastasis inhibition, with higher cell necrosis and apoptosis rates and improved survival relative to the control groups [61].

In a second study, ASO was used to silence *MALAT1* in the tumor immune microenvironment in TNBC, through subcutaneous injection in mice [65]. TP53-null mice from BALB/c mice with macrophage-enriched T12 and T11 claudin-low cells and 2208 L cells were used to investigate its action in the TNBC immune microenvironment. The ASO approach tested both alone or in combination with carboplatin and anti-PD1 therapy led to delayed tumor growth, reduced tumor volume, and prolonged survival compared to the control groups. In addition, immunohistochemistry analysis showed a decrease in the phospho-histone 3 and cleaved caspase 3 expression, leading to a reduction in proliferation, and an increase in apoptosis, respectively [65].

The last study that used ASO was conducted by Zheng et al. [66] and involved *MILIP* lncRNA. This gene plays a key role in pathogenesis by supporting protein production in TNBC through the formation of complexes with transfer RNAs (tRNAs), more specifically tRNAs for the amino acids leucine (tRNA^Leu^) and serine (tRNA^Ser^), and Eukaryotic Translation Elongation Factor 1 Alpha 1 (eEF1α1). These proteins confer sensitivity to combining *MILIP* targeting and protein synthesis inhibitors. In this study, the authors treated NOD/SCID mice-bearing MDA-MB-231.shMILIP xenografts with DOX, INN, or DOX plus INN. DOX and INN treatment resulted in higher growth inhibition of MDA-MB-231.shMILIP xenograft compared with single DOX or INN treatment. To assess the therapeutic potential of MILIP silencing, NOD/SCID mice carrying MDA-MB-231 tumors received treatment with either Gapmer.MILIP1 or Gapmer.MILIP2. Treatment with gapmers combined with INN led to inhibited tumor growth, reduced proliferation, and increased apoptosis compared to the control and gapmer-only groups [66].

#### 3.5.2. siRNAs and shRNAs

Three studies used siRNAs as an approach to silence lncRNA associated with TNBC. The lncRNA aspartyl-tRNA synthetase antisense RNA 1 siRNA (*DARS-AS1*) was cited by two articles [62,63] and the DNA-damage-inducible transcript 4 antisense RNA1 (*DDIT4-AS1*) siRNA by one article [64]. In the study of Liu et al. [63], *DARS-AS1* was pointed out as an lncRNA with a critical role in several types of tumors, including TNBC, where it is commonly overexpressed. To silence *DARS-AS1*, the authors constructed a novel nanodrug delivery system based on the EGFR-targeted aptamer CL4-modified exosomes (EXOs-CL4) for the targeted delivery of *DARS-AS1* siRNA and DOX to TNBC cells in vitro and in vivo. MDA-MB-231 tumor-bearing mouse models were established and intravenously injected with four treatment combinations: NC RNA@EXOs, *DARS-AS1* siRNA@EXOs-CL4sc (scrambled—negative control), *DARS-AS1* siRNA@EXOs-CL4 and *DARS-AS1* siRNA/DOX@EXOsCL4. The results showed that DARS-AS1 siRNA@EXOs-CL4 and *DARS-AS1* siRNA/DOX@EXOsCL4 groups were mainly focused on tumor tissues and the expression of *DARS-AS1* was downregulated compared to the other groups [63]. Furthermore, *DARS-AS1* overexpression increased the levels of Tumor Growth Factor-β (TGF-β), SMAD Family Member 3 (SMAD3), and Anti-Autophagy-Related 5 (ATG5), and promoted the conversion from anti-light chain 3-1 (LC3-I) to LC3-II; and in addition, it also induced doxorubicin resistance by activating the TGF-β/SMAD3 signaling pathway to facilitate autophagy of TNBC cells [63].

In the Liu et al. [62]’s study, *DARS-AS1* was pointed out as a promising target for therapy of chronic unpredictable mild stress-induced (CUMS) TNBC. The authors explored the correlation between *DARS-AS1* expression and investigated the molecular mechanism by which this lncRNA promoted EMT and aggressiveness of TNBC cells both in vitro and in vivo. They showed that its overexpression significantly enhanced the migration and invasion (metastasis) of TNBC tumors by inhibiting miR-129-2-3p and upregulated Cyclin-Dependent Kinase 1 (CDK1) to activate the NF-κB/STAT3 signaling pathway both in vitro and in vivo. They also determined the potential of using exosomes (EXOs) as drug delivery carriers of *DARS-AS1* siRNA for TNBC treatment, showing that treatment with *DARS-AS1* siRNA-loaded exosomes (EXOs) substantially slowed CUMS-induced TNBC cell growth and liver metastasis. These results indicated that *DARS-AS1* represents a potential therapeutic target for metastatic TNBC, and EXOs may serve as effective siRNA delivery carriers for therapy [62].

Another study using siRNA demonstrated that the *DDIT4-AS1* lncRNA was overexpressed in TNBC, promoting proliferation, migration and invasion [64]. This occurred through the stabilization of DNA-damage-inducible transcript 4 messenger RNA (DDIT4 mRNA) by *DDIT4-AS1* which recruited AU-rich Element RNA-Binding Factor 1 (*AUF1)*, by the inhibition of mTORC1 by DDIT4, resulting in the activation of autophagy. The authors designed a nano-based system called PTX@MOF/si*DDIT4-AS1*, to co-deliver PTX and *DDIT4-AS1* siRNA to TNBC tumors. This system silenced *DDIT4-AS1* expression and exerted anti-tumor activity, demonstrating an effective anti-tumor activity [64].

Finally, two articles used shRNA as an approach to silence lncRNAs. The study conducted by Dong et al. [60] suggested that the *ABHD11-AS1* lncRNA can be a potential treatment target for TNBC, considering that it is highly expressed in these tumors. TNBC mice xenografts model was established and tested the silencing of the *ABHD11-AS1* by shRNA1. The results revealed that this approach significantly inhibited the EMT markers, N-Cadherin and Snail expression and upregulated E-Cadherin, as well as miR-199a-5p expression [60]. The other study using shRNA silenced *FBXL19-AS1* [38]. The mechanism of action identified involved the overexpression of miR-378a-3p and consequently the negative regulation of the Ubiquitin Protein 2 (*OTUB2*), which is associated with TNBC progression. To validate the effects of *FBXL19-AS1*/miR-378a-3p/*OTUB2* regulatory axis in vivo, the authors transfected MDA-MB-231 cells with sh*FBXL19-AS1* and observed a reduction in the tumor volume and weight in the transfected cells compared to the control group [38].

The safety and biocompatibility of the lncRNA-targeted compounds were tested in TNBC in five of the studies selected in this review [60,61,62,63,64]. The studies conducted by He et al. [61], Liu et al. [63] and Jiang et al. [64] did not report any accumulation of the compounds studied in larger organs (heart, liver, spleen, kidney and lung) of the animals. Only the study by Liu et al. [62], with *DARS-AS1* blockade, reported histopathological alterations in the mice livers. Interestingly, that the lncRNA-associated compounds in the animals treated in these studies did not cause weight change [36,56,57,58,59], and in the study of He et al. [61], it was reported that there was a longer overall survival in comparison with the controls. These findings indicate an overall favorable safety profile and tumor-specific accumulation of the lncRNA-targeted compounds used.

Finally, only one study [67] selected in this review tested circRNAs as a target approach for TNBC therapy (Table 2, Figure 3). This subclass of ncRNAs, characterized by their circular structure [111], has been implicated in the development of TNBC progression [12]. In this study, it was shown that the *circDUSP16* promotes TNBC cell proliferation, migration, and invasion by sponging miR-1224-3p, which regulates the expression of the transcription factor dimerization partner (*TFDP2*) [67]. The authors established a xenograft tumor model by subcutaneously injecting MDA-MB-231 cells into female nude mice. The tumor volume and weight in the *circDUSP16* knockdown group were significantly lower than those in the control (si-NC) group. Additionally, the transcription levels of *circDUSP16*, *TFDP2*, *Cyclin D1*, Cyclin-Dependent Kinase 4 (*CDK4)*, and Ki67 genes were significantly decreased, while the expression of miR-1224-3p was significantly increased, indicating that *circDUSP16* knockdown inhibits the growth of xenograft tumors [67].

Collectively, the lncRNA and circRNA studied in the pre-clinical models of this systematic review highlight their role in the progression, migration and invasion of TNBC cells. Most importantly, they support their TNBC therapeutic potential, demonstrating effective tumor suppression, minimal systemic toxicity, and effective co-delivery systems.

### 3.6. Limitations of ncRNA-Based Therapies and Delivery Mechanisms

As discussed in this review, accumulating evidence demonstrates that ncRNAs play a fundamental role in regulating genes involved in critical tumor-related processes, such as proliferation, apoptosis, migration, and invasion, highlighting their potential as therapeutic targets [10,11,12]. However, several important limitations still hinder the clinical translation of these molecules in cancer therapy, particularly in TNBC.

The specificity of ncRNAs represents a key limitation, considering that a single ncRNA can regulate multiple genes and biological processes, potentially leading to off-target effects and toxicity in normal tissue/cells [112]. Studies by Dong et al. [60] and He et al. [61] highlight the specificity of lncRNAs and miRNAs as a limitation due to the modulation of poorly characterized and insufficiently studied targets, indicating the need of further investigations. Another limitation reported by He et al. [61] is related to the structural features of ncRNAs. For example, siRNAs may adopt secondary structures that hinder or limit their association with other molecules, thereby impairing target recognition. In contrast, antisense oligonucleotides (ASOs), due to their single-stranded nature, are susceptible to enzymatic degradation, limiting their direct use and requiring encapsulation for effective target delivery [61]. Overall, ncRNAs exhibit limited stability in vivo and may be degraded before reaching their intended intracellular targets [113].

Administration routes and delivery mechanisms represent additional limitations to the therapeutic application of ncRNAs identified in this review. Several strategies have been investigated and optimized to deliver inhibitors or mimics into cancer cells, either alone or in combination with other molecules. Intra-tumoral and intraperitoneal injections are widely used considering their ability to directly reach the target tissue. In the studies included in this review [36,38,43,45,48,51,52,53,54,57,59,60,67], these routes were effective in modulating the tumor microenvironment. However, it is well-recognized that these injection routes alone do not fully address challenges related to intracellular uptake and subcellular distribution of ncRNAs [113]. Intravenous administration was the second-most reported route [34,35,44,50,58], with all studies building nanotechnologies to deliver the ncRNAs. Notably, none of these studies showed adverse events or biodistribution issues. Subcutaneous administration was the least frequently reported delivery route in this review [49,65]. Limitations associated with this route may be related to the lack of assurance that ncRNAs reach the tumor site at therapeutically effective concentrations, due to restricted absorption and transport through systemic circulation. Migration of oligonucleotides to off-target sites may reduce the effective dose at the therapeutic site and potentially induce toxicity in distant tissues [114]. Nevertheless, in the study by Adewunmi et al. [65], the ASO used to silence the lncRNA *MALAT1* was successfully delivered to the tumor site in mice, resulting in significant anti-tumor effects. These findings indicate that both inhibitory molecules and administration routes require further investigation, particularly regarding off-target effects beyond the tumor site/microenvironment.

One of the limitations of this review was that several studies did not evaluate the biodistribution of the interventions nor the adverse events in vivo [36,47,53,54,55,56,59]. Biodistribution profiles are essential to determine whether nanocarriers reach their target sites at therapeutic concentrations, while avoiding accumulation in off-target tissues that could result in toxicity [115]. The physicochemical properties of nanoparticles influence how they traverse biological barriers, interact with plasma proteins, and are cleared by the reticuloendothelial system (RES), particularly in organs such as the liver and spleen [116]. Without rigorous biodistribution testing, it is not possible to confirm that a proposed nanocarrier will deliver the drug efficiently to the target sites.

Synergistic therapeutic strategies were frequently reported in this review. The therapeutic effects of ncRNAs can be enhanced when combined with conventional chemotherapeutic agents, target-specific drugs, or herbal compounds, enhancing anti-tumor efficacy while reducing the toxicity of chemotherapeutic agents when administered within nanocomplexes. He et al. [61] demonstrated that the synergy between antago3 and curcumin was a key factor in inhibiting cell proliferation and migration/invasion, as well as in effectively inducing apoptosis in MDA-MB-231 cells. This synergy was achieved using multicomponent, self-assembled polyelectrolyte nanocomplexes based on bioactive polyelectrolytes of hyaluronic acid and chitosan hydrochloride, which exhibit good biocompatibility and biodegradability. These findings reinforce the need for drug delivery systems based on biologically compatible nanoparticles to enhance ncRNA delivery and therapeutic efficacy [61,62].

Exosomes are extracellular nanovesicles that have been used as natural drug delivery carriers for biological macromolecules via endocytosis due to their nanoscale size, excellent biocompatibility, presence of specific receptors, and lack of immunogenicity and inflammatory properties [117]. In studies by Liu et al. [62,63], *DARS-AS1* siRNA was loaded onto exosomes (EXOs) using a modified calcium-mediated transfection method, which successfully delivered the siRNA to the TNBC tissue, acting synergistically with or without doxorubicin. Additionally, Liu et al. [40] employed cancer cell–platelet fusion membrane vesicles (CPMVs) loaded with miR-10b and miR-21, demonstrating good biocompatibility and immunomodulatory capacity. The authors noted, however, that nanoparticle and cell membrane (CM)-camouflaged drug delivery vehicles still face challenges, such as opsonization and low tissue-targeting efficiency, which may limit their clinical potential. Other nanotechnology-based methodologies used in this review for the effective delivery of ncRNAs to TNBC tissues include specially designed nanocapsules, such as liposomes, polymer-based nanoparticles, and inorganic nanoparticles to ensure efficient target engagement [64]. Jiang et al. [64] employed a core–shell metal–organic framework (MOF) nanosystem for the synergistic co-administration of *DDIT4-AS1* siRNA and paclitaxel, suggesting that the “*DDIT4-AS1/DDIT4*/autophagy” axis may represent a promising new therapeutic target for TNBC. Liu et al. [41] utilized gold nanodots and nanostars surface-coated with chitosan-β-cyclodextrin using a microfluidic-optimized method and urokinase plasminogen activator (uPA) peptide, to co-deliver “suicide” genes (thymidine kinase–p53–nitroreductase: TK-p53-NTR) and therapeutic miRNAs (anti-miR-21, anti-miR-10b, and miR-100) in lung cancer metastasis. Although positive results were reported, the authors emphasized the need for larger experimental animal cohorts and dose optimization. Chen et al. [42] developed a biocompatible nanosystem based on amphiphilic phosphorus dendrimer micelles (1-C12G1) to co-deliver miR-21 inhibitors (miR-21i) and doxorubicin for combination therapy in TNBC. This co-delivery system exhibited efficiency when internalized by cancer cells, enabling synergistic anticancer effects through complementary mechanisms.

### 3.7. Future Perspectives of ncRNA-Based Therapies in TNBC

Challenges related to the delivery of ncRNAs, whether alone or in combination with other therapeutic agents, remain a major barrier to their clinical application in TNBC treatment. Further studies should therefore focus on the rational selection of molecular targets, optimize delivery strategies, administration routes and biodistribution to minimize off-target effects, and enhance therapeutic precision.

Nevertheless, numerous innovative technologies are currently being developed to overcome these limitations. The development of next-generation delivery systems capable of efficient tumor targeting, cell membrane translocation, and controlled intracellular release will be critical. Stimuli-responsive and biomimetic nanocarriers, including exosome-based platforms, hold promises for improving stability, biodistribution, and safety profiles. In parallel, rational combination strategies integrating ncRNAs with chemotherapy, targeted agents, or immunotherapy may potentiate anti-tumor efficacy and help overcome therapeutic resistance. These strategies may support the development of structured and translatable ncRNA-based clinical protocols, emerging as novel frontier for more effective, specific, and personalized therapies in the treatment of TNBC.

## 4. Conclusions

This review highlighted the central role of ncRNAs, including miRNAs, lncRNAs, and circRNAs, as key molecules regulators of the pathogenesis, progression, and treatment responsiveness of TNBC. Although significant challenges remain and additional pre-clinical studies are required before ncRNA-based therapies can be translated into clinical practice, particularly with respect to optimizing delivery platforms and ensuring safety and specificity, the compelling in vivo efficacy data presented here supports ncRNAs as critical molecular targets for TNBC treatment. These findings highlight the substantial potential of ncRNAs for the development of new and effective treatment modalities for this aggressive subtype of breast cancer, offering a promising avenue to overcome the limitations of current therapeutic approaches.

## Figures and Tables

**Table 1:** MiRNAs identified with therapeutic potential in TNBC and their corresponding gene targets, therapeutic strategy, delivery system, and tumor effect.

miRNA	Target-Genes	Therapeutic Approach	Therapeutic Agent	Delivery System	Tumor Effect	References
miR-10b	*PDCD4* [40], *HOXD10* [41], *PTEN* [41]	AntagomiR	Doxorubicin [40], Ganciclovir [41], CB1954 [41], TK-p53-NTR triple therapeutic gene [41]	Cancer cell–platelet membrane fusion nanovesicle [40], gold nanoparticles [41]	Inhibited tumor growth [40,41], improved chemotherapy sensitivity and response [40], improved survival rates [41]	[40,41]
miR-21	*PDCD4* [40], *HOXD10* [41] *PTEN* [41,42], *BAX* [42], p53 [42]	AntagomiR [33,40,41], miR-inhibitor [42]	Doxorubicin [40,42], Ganciclovir [33,41], CB1954 [33,41], TK-p53-NTR triple therapeutic gene [33,41]	Cancer cell–platelet membrane fusion nanovesicle [40], gold nanoparticles [41], PLGA-PEG-PEI polymer nanoparticle [33], and amphiphilic phosphorous dendron micelles nanosystem [42]	Promoted chemotherapy sensitivity [40], improved therapeutic response [40], increased survival rates [33,40,41], inhibited tumor growth [41,42], enhanced apoptosis [33], reduced systemic toxicity to chemotherapy [42], increased expression of apoptosis-associated proteins [42]	[33,40,41,42]
miR-22	NA	AntagomiR	NA	Intraperitoneal injection	Inhibited tumor growth and proliferation	[43]
miR-34a	*BCL-2* [34,39,44], *PDL-1* [44], *CD44* [34,37], *WNT7b* [37], *TCF4* [37], *ADLH1A3* [37]	miRNA-mimic	Doxorubicin [37], Pyropheophorbide-a [39]	Metal–organic framework nanoparticles/intravenous injection [44], intra-tumoral injection [45], nonviral vector PEI-SPDP-Man/intravenous injection [34], mesoporous silica nanoparticles [37], and aptamer-modified liposome [39]	Enhanced and induced cancer cell apoptosis [44,45], inhibited tumor growth [44,45], proliferation [34,37], metastasis [39] and immune escape [39]	[34,37,39,44,45]
miR-100	*HOXD10* [41], *PTEN* [41]	miRNA-mimic	CB1954 [33,41] Ganciclovir [33,41], and TK-p53-NTR triple therapeutic gene [33,41]	Gold nanoparticle [41], PLGA-PEG-PEI polymer nanoparticle [33]	Enhanced apoptosis [41], reduced tumor growth [33,41], increased survival rates [33,41]	[33,41]
miR-105-5p	*HDAC2*, *FOXG1*, *BCL-2*, *BAX*	miRNA inhibitor	NA	Lentivirus/tail vein injection	Inhibited proliferation and tumor growth, induced apoptosis	[46]
miR-139	*SOX8*	miRNA-mimic	NA	NI	Inhibited tumor growth	[47]
miR-143	*HK2*	miRNA-mimic	Gemcitabine	Intra-tumoral injection	Inhibited tumor growth, reduced tumor resistance to chemotherapy	[48]
miR-146b-5p	*DGCR8*, *FOXO1*, *METTL14*, *SCAF11*	miRNA-mimic	Cold atmospheric plasma	Subcutaneous injection on tumor site	Reduced tumor size	[49]
miR-155	*CD80*, *CD86*, MHC II [35], *HMGB1* [50]	miRNA-mimic	Curcumin [50]	PEG-CDM-PEI[Man]-ox-PCL nanocomplex/intravenous injection [35], reactive oxygen species/glutathione dual-sensitive nanoparticle/tail vein injection [50]	Inhibited tumor growth [35,50] and metastasis [35,50], generated anti-tumor immune response [35,50], induced cell apoptosis [35,50], prolonged survival rates [35,50]	[35,50]
miR-155-5p	Cleaved caspase-1, *EGFR*, *GSDME*	AntagomiR	Cetuximab	Intraperitoneal injection	Inhibited tumor growth and proliferation, enhanced anti-tumor effect of chemotherapeutic, induced cell apoptosis and pyroptosis	[51]
miR-182-5p	*NOTCH1*	AntagomiR	NA	Intra-tumoral injection	Inhibited tumor growth and proliferation	[52]
miR-199a-5p	*ZEB1*	Vesicular stomatitis virus expressing pre-miR-199a-5p	NA	Vesicular stomatitis virus	No effect	[53]
miR-320c	*Chk1*, *γ-H2AX*, cleaved caspase-3	miRNA-mimic	Oxaliplatin	Intra-tumoral injection	Inhibited tumor growth, increased apoptosis and treatment response	[54]
miR-325-3p	*GSTP1*	miRNA-mimic	Paclitaxel	NI	Inhibited tumor growth and proliferation, induced ferroptosis, restored chemotherapy sensibility	[55]
miR-326	*FN1/ITGA5* receptor, *HIF-1α*, *FAK*/*SRC* pathway	Doxycycline miRNA (induction)	Doxorubicin	NI	Inhibited tumor growth, potentiated chemotherapy response	[56]
miR-506	*ERK/FOS* pathway, E-Cadherin, N-Cadherin, Snail, *PENK*, *VIM*	miRNA-mimic	NA	Gelatin Nanospheres/Intra-tumoral injection	Inhibited tumor growth, invasion ability and metastasis, promoted necrosis and apoptosis	[57]
miR-588	*CCL5*	miRNA-mimic	NA	cRGD-modified exosome/Tail vein injection	Inhibited tumor growth, remodeled immunosuppressive tumor microenvironment	[58]
miR-606	*BAX*, *BCL-2*, *PCNA*, *STC1*	miRNA-mimic	NA	Intra-tumoral injection	Inhibited tumor growth and metastasis	[36]
miR-6162	*GRP78/AKT* axis, *HIF1α*, *GLUT1*, *LDHA*, *PTEN*	miRNA-mimic	NA	Intra-tumoral injection	Inhibited tumor growth and metastasis, induced apoptosis	[59]

ATP: Adenosine triphosphate; *ADLH1A3*: Aldehyde Dehydrogenase 1 Family Member 3; *BAX*: BCL2-Associated X; *BCL*-2: B-Cell Lymphoma 2; *CASP1*: Caspase 1; *CCL5*: C-C Motif Chemokine Ligand 4; *CD44*: Cluster of Differentiation 44; *CD80*: Cluster of Differentiation 80; *CD86*: Cluster of Differentiation 86; *Chk1*: Checkpoint Kinase 1; *DGCR8*: Microprocessor Complex Subunit DGCR8; E-Cadherin: Epithelial Cadherin; *EGFR*: Epidermal Growth Factor Receptor; *ERK/FOS*: Extracellular Signal-Regulated Kinase/FOS Proto-Oncogene; *FAK/Src*: Focal Adhesion Kinase/v-src Sarcoma Viral Oncogene Homolog; *FOXG1*: Forkhead Box Protein G1; *FOXO1*: Forkhead Box 1; *FN1/ITGA5*: Fibronectin 1/Integrin Subunit Alpha 5; *GLUT1*: Glucose Transporter Type 1; *GRP78/Akt axis*: Glucose-Regulated Protein 78/Protein Kinase B; *GSDME*: Gasdermin E; *GSTP1*: Glutathione S-Transferase P 1; *HDAC2*: Histone Deacetylase 2; *HIF1α*: Hypoxia-Inducible Factor 1-Alpha; *HK2*: Hexokinase 2; *HMGB1*: High Mobility Group Box 1; *HOXD10*: Homeobox D10; *LDHA*: Lactate Dehydrogenase A; *METTL14*: Methyltransferase 14; MHC II: Major Histocompatibility Complex Class II; NA: not applied; N-cadherin: Neural Cadherin; NI: not informed; *NOTCH1*: Neurogenic Locus Notch Homolog Protein 1; *PCNA*: Proliferating Cell Nuclear Antigen; *PDCD4*: Programmed Cell Death 4 Protein; *PDL-1*: Programmed Cell Death Ligand 1; *PENK*: Proenkephalin; *PLK1*: Polo-like Kinase 1; *PTEN*: Phosphatase and TENsin Homolog Deleted on Chromosome 10; *SCAF11*: SR-Related CTD-Associated Factor 11; *SOX8*: SRY-Box Transcription Factor 8; *STC1*: Repressing Stanniocalcin 1; *TCF4*: Transcription Factor 4; *VIM*: Vimentin; *ZEB1*: Zinc Finger E-Box-Binding Homeobox 1; *WNT7b*: Wnt Family Member 7B1; *γ-H2AX*: Phosphorylated Histone H2AX.

**Table 2:** LncRNAs and circRNA identified with therapeutic potential in TNBC and their corresponding gene targets, therapeutic strategy, delivery system and tumor effect.

ncRNA	Target-Genes	Therapeutic Approach	Therapeutic Agent	Delivery System	Tumor Effect	References
*ABHD11-AS1*	E-Cadherin N-Cadherin, Snail, miR-199a-5p	shRNA	NA	Intra-tumoral injection	Suppressed tumor growth	[60]
*ASBEL*	*BCL-2*, *BTG3, c-MET*	ASO	Curcumin	Polyelectrolyte nanocomplex	Suppressed tumor growth and metastasis, promoted apoptosis, extended survival rate	[61]
*DARS-AS1*	*CDK1* [62], miR-129-2-3p [62], *SMAD3* [62], *ATG5* [63], *TGF-β* [63]	siRNA	Doxorubicin [63]	Exosome [62] CL4-modified exosomes [63]	Suppressed tumor growth [62,63], metastasis [63], and autophagy [63], decreased doxorubicin resistance [62], induced apoptosis [62,63]	[62,63]
*DDIT4-AS1*	*AUF1*, *DDIT4* mRNA	siRNA	Paclitaxel	Core–shell nanoparticle system	Suppressed tumor growth and proliferation, enhanced sensitivity to chemotherapeutic	[64]
*FBXL19-AS1*	miR-378a-3p, *OTUB2*	shRNA	NA	Intra-tumoral injection	Suppressed tumor growth and proliferation	[38]
*MALAT1*	Immunosuppressive myeloid cells	ASO	Carboplatin	Subcutaneous injection	Reduced tumor growth and proliferation, improved response to chemotherapy, promoted apoptosis	[65]
*MILIP*	*eEF1α1*, type II tRNAs (tRNA^Leu^ and tRNA^Ser^)	ASO	Doxorubicin, Omacetaxine mepesuccinate (INN)	NI	Retarded tumor growth, decreased proliferation, enhanced chemotherapy effect and apoptosis	[66]
*circDUSP16*	*TFDP2*, miR-1224-3p, Cyclin D1, *CDK4*, Ki67	siRNA	NA	Intra-tumoral injection	Suppressed tumor growth	[67]

*ATG5*: Anti-Autophagy-Related 5; *AUF1*: AU-Rich Element RNA-Binding Factor 1; *BCL-2*: B-Cell Lymphoma 2; *BTG3*: B-Cell Translocation Gene 3; *CDK1*: Cyclin-Dependent Kinase 1; *CDK4*: cyclin-Dependent Kinase 4; *c-MET*: Cellular Mesenchymal–EpithelialTransition Gene; *DDIT4 mRNA*: DNA-Damage-Inducible Transcript 4 Messenger RNA; E-Cadherin: Epithelial Cadherin; *eEF1α1*: Eukaryotic Translation Elongation Factor 1 Alpha 1; *Ki67*: Antigen Kiel 67; NA: not applied; N-Cadherin: Neural Cadherin; NI: not informed; *OTUB2*: Ubiquitin Protein 2; *SMAD3*: SMAD Family Member 3; *TFDP2*: Transcription Factor Dimerization Partner; *TGF-β*: Transforming Growth Factor-Beta; tRNA^Leu^: Transfer RNA for the amino acid leucine; tRNA^Ser^: Transfer RNA for the amino acid serine; tRNAs: transfer RNAs.

## Data Availability

All the data is presented in this article.

## Supplementary Materials

The following supporting information can be downloaded at https://www.mdpi.com/article/10.3390/ijms27041882/s1.
