# Supplementary material for: Non-Coding RNA-Based Therapeutic Strategies in Triple-Negative Breast Cancer: A Systematic Review
Source: Int J Mol Sci. 2026 Feb 15;27(4):1882. doi: 10.3390/ijms27041882 (PMC12940445; doi:10.3390/ijms27041882)
Supplement: Supplementary file 1 [file ijms-27-01882-s001.zip › ijms-4074506-SI.pdf]

Targeting ncRNAs in Triple-Negative Breast Cancer: Pre-Clinical Evidence from In Vivo Tumor Models
Supplementary Material

Table S1. Complete searched terms used in each database.

Database	Search string	Date Range
PubMed	[(Untranslated RNA OR npcRNA OR RNA, Nontranslated OR Nontranslated RNA OR RNA, Non-Peptide-Coding OR Non-Peptide-Coding RNA OR RNA, Non Peptide Coding OR RNA, Non-Protein-Coding OR Non-Protein-Coding RNA OR RNA, Non Protein Coding OR RNA, Noncoding OR Noncoding RNA OR RNA, Non-Coding OR Non-Coding RNA OR RNA, Non Coding OR Enhancer RNA OR Catalytic RNA OR ribozyme OR Long Noncoding RNA OR Small Untranslated RNA OR MicroRNAs OR CRISPR-Cas RNA System OR Small kinetoplastid mitochondrial RNA OR Small Cytoplasmic RNA OR Small Interfering RNA OR Small Nuclear RNA OR Spliced Leader RNA OR Untranslated Regions OR 3' Untranslated Regions OR 5' Untranslated Regions OR Regulatory RNA Region) AND (therapy OR treatment OR disease management) AND (ER-Negative PR-Negative HER2-Negative Breast Neoplasms OR ER Negative PR Negative HER2 Negative Breast Neoplasms OR Triple-Negative Breast Cancer OR Breast Cancer, Triple-Negative OR Breast Cancers, Triple-Negative OR Triple-Negative Breast Cancers OR Triple-Negative Breast Neoplasm OR Breast Neoplasm, Triple-Negative OR Breast Neoplasms, Triple-Negative OR Triple Negative Breast Neoplasm OR Triple-Negative Breast Neoplasms OR ER-Negative PR-Negative HER2-Negative Breast Cancer OR ER Negative PR Negative HER2 Negative Breast Cancer OR Triple Negative Breast Cancer) AND (in vivo OR clinical trial)] NOT review	2020 - 2025
BVS	(Triple Negative Breast Neoplasms) AND (RNA, Untranslated OR RNA, Long Noncoding OR MicroRNAs OR RNA, Circular) AND (treatment) NOT (review)	2020 -2025
Embase	('untranslated rna'/exp OR 'ribozyme'/exp OR 'long untranslated rna'/exp OR 'microrna'/exp OR 'small untranslated rna'/exp OR 'crispr-cas system guide rna'/exp OR 'small cytoplasmic rna'/exp OR 'small interfering rna'/exp OR 'small nuclear rna'/exp OR 'spliced leader rna'/exp OR 'untranslated region'/exp) AND 'therapy'/exp AND 'triple negative breast cancer'/exp AND ('in vivo study' OR 'clinical study') NOT 'review'	2020-2025

Table S2. Risk of Bias using SYRCLE's Risk of Bias Tool.

Study	D1	D2	D3	D4	D5	D6	D7	D8	D9	D10
Liu et al., 2022 [41]	U	U	Y	U	U	U	U	Y	U	Y
Liu et al., 2022 [41]	U	Y	U	U	U	U	U	Y	U	Y
Kumar et al., 2021 [33]	U	Y	U	U	U	U	U	U	U	Y
Chen et al., 2023 [42]	Y	Y	U	Y	Y	Y	Y	U	U	Y
Panella et al., 2023 [43]	U	U	U	Y	Y	U	Y	U	Y	N
Zhao et al., 2021 [44]	U	U	Y	U	Y	Y	Y	Y	U	Y
Deng et al., 2023 [45]	U	Y	Y	U	U	Y	U	Y	Y	Y
Han et al., 2023 [34]	U	U	Y	U	U	U	U	U	U	Y
Duan et al., 2024 [37]	U	Y	U	U	U	U	U	U	U	Y
Xiong et al., 2025 [39]	U	U	U	U	U	U	U	U	U	Y
Wang et al., 2025 [46]	Y	Y	Y	Y	Y	Y	Y	Y	Y	Y
Dong et al., 2020 [47]	Y	Y	Y	Y	Y	Y	Y	U	U	U
Xi et al., 2022 [48]	Y	Y	U	Y	Y	Y	Y	U	Y	Y

Study	D1	D2	D3	D4	D5	D6	D7	D8	D9	D10
Dai et al., 2025 [49]	Y	Y	Y	Y	Y	Y	Y	Y	Y	U
Jing et al., 2023 [35]	U	U	U	U	U	U	U	U	Y	Y
Li et al., 2024 [50]	Y	Y	Y	Y	Y	Y	Y	Y	Y	Y
Xu et al., 2021 [5]	Y	Y	Y	Y	Y	Y	Y	Y	Y	Y
Li et al., 2024 [52]	U	Y	U	Y	Y	Y	Y	U	U	Y
St-Cyr et al., 2025 [53]	U	Y	U	Y	Y	Y	Y	Y	U	Y
Lim et al., 2020 [54]	U	Y	U	Y	Y	U	Y	U	Y	Y
Wang et al., 2025 [46]	Y	Y	Y	Y	Y	Y	Y	Y	U	Y
Assidicky et al., 2022 [56]	U	Y	U	Y	Y	Y	Y	U	U	N
Liu et al., 2021 [57]	U	Y	Y	Y	Y	Y	Y	Y	Y	Y
Zhang et al., 2024 [58]	Y	Y	Y	U	Y	Y	Y	U	Y	Y
Choi et al., 2024 [36]	U	Y	U	U	U	U	U	U	U	Y
Wang et al., 2025 [55]	Y	Y	Y	Y	Y	Y	Y	U	Y	Y

Study	D1	D2	D3	D4	D5	D6	D7	D8	D9	D10
Dong et al., 2023 [60]	Y	Y	Y	Y	Y	Y	Y	Y	U	U
He et al., 2023 [61]	Y	Y	Y	Y	Y	Y	Y	Y	Y	Y
Liu et al., 2022 [62]	Y	Y	Y	Y	Y	Y	Y	Y	Y	Y
Liu et al., 2023 [92]	Y	Y	Y	Y	Y	Y	Y	Y	Y	Y
Jiang et al., 2023 [64]	Y	Y	U	Y	Y	Y	Y	U	U	Y
Guo et al., 2024 [38]	Y	Y	U	U	U	U	U	U	U	U
Adewunmi et al., 2023 [65]	Y	Y	U	Y	Y	Y	Y	Y	Y	U
Zheng et al., 2024 [66]	Y	Y	U	Y	Y	Y	Y	U	U	U
Wei et al., 2025 [67]	Y	Y	U	Y	Y	Y	Y	Y	U	Y

Y= Yes (low risk of bias). N = No (High risk of bias). U = Unclear (unclear risk of bias). Domains of SYRCLE's Risk of Bias Tool: D1: Was the allocation sequence adequately generated and applied? D2: Were the groups similar at baseline or were they adjusted for confounders in the analysis? D3: Was the allocation to the different groups adequately concealed during? D4: Were the animals randomly housed during the experiment? D5: Were the caregivers and/or investigators blinded from knowledge which intervention each animal received during the experiment? D6: Were animals selected at random for outcome assessment? D7: Was the outcome assessor blinded? D8: Were incomplete outcome data adequately addressed? D9: Are reports of the study free of selective outcome reporting? D10: Was the study apparently free of other problems that could result in high risk of bias?

Table S3. Title, authorship, country of study, and year of publication of the 34 articles selected

Title	Authors	Country	Year	#
Inhibition of Chk1 by miR-320c increases oxaliplatin responsiveness in triple-negative breast	Lim et al.	South Korea	2020	[54]

Title	Authors	Country	Year	#
cancer				
MicroRNA-139 suppresses the tumorigenicity of triple negative breast cancer cells by targeting sox8	Dong et al.	China	2020	[47]
Camouflaged Hybrid Cancer Cell-Platelet Fusion Membrane Nanovesicles Deliver Therapeutic MicroRNAs to Presensitize Triple-Negative Breast Cancer to Doxorubicin	Liu et al.	USA	2021	[40]
Downregulation of miR-155-5p enhances the anti-tumor effect of cetuximab on triple-negative breast cancer cells via inducing cell apoptosis and pyroptosis.	Xu et al.	China	2021	[51]
Dual Roles of Metal-Organic Frameworks as Nanocarriers for miRNA Delivery and Adjuvants for Chemodynamic Therapy.	Zhao et al.	China	2021	[44]
miR-506-loaded gelatin nanospheres target PENK and inactivate the ERK/Fos signaling pathway to suppress triple-negative breast cancer aggressiveness.	Liu et al.	China	2021	[57]
Ultrasound Triggered Co-Delivery of Therapeutic MicroRNAs and a Triple Suicide Gene Therapy Vector by Using Biocompatible Polymer Nanoparticles for Improved Cancer Therapy in Mouse Models	Kumar et al.	USA	2021	[33]
Combination treatment with hENT1 and miR-143 reverses gemcitabine resistance in triple-negative breast cancer	Xi et al.	China	2022	[48]
Exosomes deliver lncRNA DARS-AS1 siRNA to inhibit chronic unpredictable mild stress-induced TNBC metastasis.	Liu et al.	China	2022	[62]
Inhaled Gold Nano-star Carriers for Targeted Delivery of Triple Suicide Gene Therapy and Therapeutic MicroRNAs to Lung Metastases: Development and Validation in a Small Animal Model	Liu et al.	USA	2022	[41]
Targeting HIF1-alpha/miR-326/ITGA5 axis potentiates chemotherapy response in triple-negative breast cancer.	Assidicky et al.	USA	2022	[56]
Amphiphilic phosphorous dendron micelles co-deliver microRNA inhibitor and doxorubicin for augmented triple negative breast cancer therapy.	Chen et al.	China	2023	[42]
CL4-modified exosomes deliver lncRNA DARS-AS1 siRNA to suppress triple-negative breast cancer progression and attenuate doxorubicin resistance by inhibiting autophagy	Liu et al.	China	2023	[63]

Title	Authors	Country	Year	#
Coordinated modulation of long non-coding RNA ASBEL and curcumin co-delivery through multicomponent nanocomplexes for synchronous triple-negative breast cancer theranostics	He et al.	China	2023	[61]
Efficient TNBC immunotherapy by dual reprogramming tumor-infiltrating dendritic cells and tumor-associated macrophages with stimulus-responsive miR155 nanocomplexes	Jing et al.	China	2023	[35]
Knockdown of ABHD11-AS1 prevents the procession of TNBC by upregulating miR-199a-5p	Dong et al.	China	2023	[60]
p53 downregulates PD-L1 expression via miR-34a to inhibit the growth of triple-negative breast cancer cells: a potential clinical immunotherapeutic target	Deng et al.	China	2023	[45]
Polymer-Initiating Caveolae-Mediated Endocytosis and GSH-Responsive MiR-34a Gene Delivery System for Enhanced Orthotopic Triple Negative Breast Cancer Therapy.	Han et al.	China	2023	[34]
Targeted Inhibition of lncRNA Malat1 Alters the Tumor Immune Microenvironment in Preclinical Syngeneic Mouse Models of Triple-Negative Breast Cancer	Adewunmi et al.	USA	2023	[65]
Targeting lncRNA DDIT4-AS1 Sensitizes Triple Negative Breast Cancer to Chemotherapy via Suppressing of Autophagy	Jiang et al.	China	2023	[64]
Targeting of microRNA-22 Suppresses Tumor Spread in a Mouse Model of Triple-Negative Breast Cancer	Panella et al.	USA	2023	[43]
Engineered Exosomes Carrying miR-588 for Treatment of Triple Negative Breast Cancer Through Remodeling the Immunosuppressive Microenvironment.	Zhang et al.	China	2024	[58]
Exosomal miR-182-5p from breast cancer cells reprogram tumor-associated macrophages and promote triple-negative breast cancer progression by targeting Notch1 in macrophages	Li et al.	China	2024	[52]
Exploring the regulatory role of FBXL19-AS1 in triple-negative breast cancer through the miR-378a-3p/OTUB2 axis.	Guo et al.	China	2024	[38]
Homologous Tumor Cell-Derived Biomimetic Nano-Trojan Horse Integrating Chemotherapy with Genetherapy for Boosting Triple-Negative Breast Cancer Therapy	Duan et al.	China	2024	[37]
MicroRNA-606 inhibits the growth and metastasis of triple-negative breast cancer by targeting Stanniocalcin 1.	Choi et al.	South Korea	2024	[36]
MILIP Binding to tRNAs Promotes Protein Synthesis to Drive Triple-Negative Breast Cancer	Zheng et al.	China	2024	[66]

Title	Authors	Country	Year	#
Reactive oxygen species/glutathione dual sensitive nanoparticles with encapsulation of miR-155 and curcumin for synergized cancer immunotherapy	Li et al.	China	2024	[50]
CircDUSP16 mediates the effect of triple-negative breast cancer in pirarubicin via the miR-1224-3p/TFDP2 axis.	Wei et al.	China	2025	[67]
Cold atmospheric plasma targets triple negative breast cancer cells via SCAF11-mediated competitive protein degradation and synergizes with miRNA-146b-5p.	Dai et al	China	2025	[49]
miR-6126 modulates GRP78 to suppress the Warburg effect and mitochondrial dynamics in triple-negative breast cancer	Wang et al.	China	2025	[59]
miRNA-105–5p regulates the histone deacetylase HDAC2 through FOXG1 to affect the malignant biological behavior of triple-negative breast cancer cells	Wang et al.	China	2025	[46]
Photoswitchable dynamics and RNAi synergist with tailored interface and controlled release reprogramming tumor immunosuppressive niche.	Xiong et al.	China	2025	[39]
Targeting the miR-325-3p/GSTP1 axis overcomes paclitaxel resistance in triple-negative breast cancer by inducing ferroptosis	Wang et al.	China	2025	[55]
Therapeutic limitations of oncolytic VSVd51-mediated miR-199a-5p delivery in triple negative breast cancer models	St-Cyr et al.	Canada	2025	[53]

Obs. References cited in Supplementary Materials are included in the main reference list.
